# Looking through the Keyhole: Exploring Realities and Possibilities for School Breakfast Programs in Rural Western Australia

**DOI:** 10.3390/nu10030371

**Published:** 2018-03-17

**Authors:** Simon O. Ichumar, Emma E. Dahlberg, Ellen B. Paynter, Fiona M. C. Lucey, Miranda R. Chester, Lennelle Papertalk, Sandra C. Thompson

**Affiliations:** 1 Western Australian Centre for Rural Health, The University of Western Australia, 167 Fitzgerald St Geraldton, WA 6530, Australia; simonomeke@gmail.com (S.O.I.); dahlbrg2@illinois.edu (E.E.D.); paynter.ellen@gmail.com (E.B.P.); Fiona.lucey@uwa.edu.au (F.M.C.L.); Lenny.papertalk@uwa.edu.au (L.P.); 2 Foodbank, Perth, WA 6105, Australia; Miranda.chester@foodbankwa.org.au

**Keywords:** school breakfast program, food, nutrition, health education, disadvantaged children, food insecurity, Food bank, food literacy, nutrition education intervention, vulnerable populations

## Abstract

Objective: To assess the school breakfast program (SBP) in two schools with high Aboriginal student populations in rural Western Australia, their contribution to holistic support, nutritional health education and possibilities for improvement. Methods: The operations and functioning of one regional and one remote SBP were assessed by stakeholder inquiry related to process and challenges, observations and documentary review. An intervention to increase health education, social interaction and learning about nutrition and food origins implemented in one school was assessed. Results: Strengths, system and structural factors that impeded realisation of optimal outcomes of the SBPs were identified. The SBPs focussed on serving food rather than building nutritional understanding or on social interactions and support. Systems for delivery and management of the programs largely relied on staff with limited time. When offered a more interactive and social environment, children enjoyed learning about food. Conclusions: Opportunities for SBPs to offer holistic support and educational enhancement for disadvantaged children are limited by the realities of pressures on staff to support them and a view constraining their primary role as food delivery. The lack of volunteer support in disadvantaged schools limits the potential benefits of SBPs in providing psychosocial support. Health education resources which exist for use in SBPs are not necessarily used.

## 1. Introduction

### 1.1. The Context of Health for Aboriginal and Torres Strait Islander Australians

Like other developed countries with a history of colonisation in which the original inhabitants were dispossessed of their lands with consequent disruption of their traditional culture and lifestyle, exposure to new diseases, discrimination and violence, Aboriginal and Torres Strait Islander (hereafter Indigenous) Australians have a well described life expectancy gap compared to the general Australian population [1]. It is both the size of the health disparity gap, which is worse and closing more slowly than that in developed nations with similar histories [2], and the constellation of disadvantage across many health and social indicators (such as education, housing and justice) that demand that particular attention is given to improving health and wellbeing in this special group [3]. It is important in these efforts that attention is given to both the processes needed to improve the health of Indigenous Australians and to building evidence of “what works”. Efforts that utilise approaches which maximise existing investments and opportunities are likely to result in more rapid health gain, and this arguably provides a strong rationale for evaluation of existing programs and adopting quality improvement approaches.

Burden of disease studies have identified key priorities for intervention, and highlighted the burden of chronic disease and mental health issues for Indigenous Australians. As stated by Lopez in the foreword to the Burden of Disease study, one-third of young Indigenous men aged 15 years will be dead before age 60, compared with 8% in the Australian population, a four-fold increase in risk of death, largely due to excess mortality from such causes as ischaemic heart disease, suicide and Type 2 diabetes [4]. The onset of these chronic diseases occurs many years earlier than for the non-Indigenous population, as shown in many studies [5,6,7,8,9,10]. Given the large burden of both chronic and mental health issues, there is a need for prevention efforts to be focused on children and youth and to embrace the holistic models of health that Indigenous people have repeatedly identified as consistent with their cultural values [11].

It is only relatively recently that Australia has turned serious attention to the challenges associated with ‘closing the gap’ [12]. While there has been some progress against the targets, many are not on track, including two associated with schooling: attendance at school and halving the gap in numeracy and literacy [13]. This reinforces the need for efforts to improve Indigenous education outcomes and to look critically at what has been shown to work in school settings [14] while also recognising the important impact of social determinants on health and education [3].

### 1.2. Health and Education Disparities in Australia for Indigenous Children in Rural Australia

The close nexus between educational achievement and health outcomes is well described. Adults with higher levels of education have healthier behaviors related to diet and exercise, and have a reduced likelihood of engaging in risky behaviors such as smoking and drinking [15]. Education is considered to be one of the social determinants of health and the impact of education on health behaviours likely stems from education’s impact on skills as well as socioeconomic status. Higher levels of educational attainment improve opportunities for employment and are associated with higher income and social participation.

Substantial disparities exist in school based educational outcomes between Indigenous and non-Indigenous Australians. Students in rural and remote schools in particular continue to fall short of educational benchmarks. This is despite there being a considerable body of work that has identified “what works” in improving Aboriginal education outcomes [14,16]. Included as part of Doyle and Hill’s review was the necessity for a holistic school approach, and this included provision of school breakfast programs [16].

### 1.3. Benefits of School Breakfast Programs

There is substantial evidence showing that children’s participation in a school breakfast program (SBP) is associated with a wide range of educational benefits. These include improvements in attention, memory recall, creative thinking and physical endurance [17,18]. Attendance in SBPs has also been shown to improve students’ motivation, psychological functioning, school attendance and punctuality [19,20]. SBPs also positively influence students’ on-task behavior which can result in decreased disruptions in class and an overall improvement in classroom performance [21]. Children who attend SBPs have also been shown to develop better quality relationships with their peers and experience less victimization [22]. Moreover, studies have shown that the opportunities available for children to engage in playful social interaction in schools such as during break times have decreased over the years as more emphasis is being placed on academic activities. Coupled with this have been increasing parental concerns regarding the safety of their children while out of home [23,24]. Hence, SBPs provide opportunities as places where children can develop their social interaction abilities and build useful relationships in a safe environment available in many communities and under the supervision of adults and where there could be the opportunity to gain useful life skills such as by participating in food preparation appropriate for age.

As a result of evidence of nutritional, educational and psychosocial benefits, SBPs now operate in many countries to address the nutrition, health, local child care and education needs of diverse school communities [25,26,27]. The objectives of different SBPs have varied, some focusing on the provision of a healthy breakfast while others incorporate elements of health education, childcare and encouraging informal interaction between children and school staff so as to foster psychosocial nourishment [25,26,27].

### 1.4. Background to School Breakfast Programs in Western Australia

SBPs were established in Western Australia in response to multiple drivers. Studies had showed that between 10% to 17% of primary school students and 29% to 38% of secondary students in Western Australia (WA) do not consume breakfast on a daily basis [28]. The reasons given for failure to have regular breakfast included those that catalysed initiation of SBPs elsewhere: lack of food in the household; lack of parent time; children not being hungry first thing in the morning; no adult to prepare the breakfast as parents leave early for work, children lacking food preparation/cooking skills; time constraints with having to leave home early in the morning; and children not wanting to eat alone [29,30].

To help counter omission of breakfast by students, Foodbank WA established the *School Breakfast Program* to support schools throughout WA to provide free breakfast and emergency meals to students. Operating within Foodbank WA’s *Healthy Food for All* strategy [31], the SBP commenced in 17 schools in 2001 and by 2013 had been implemented in 426 schools throughout WA [29]. Just under half (41%) of these schools are located in the metropolitan area, with the remainder operating in regional WA, from regional cities to remote towns located hundreds of kilometers from Foodbank WA’s branch network. These SBPs reach over 16,000 children with 53,000 breakfasts and 20,600 ‘emergency’ meals served per week and provide students with breakfast within the school premises in an environment that is safe and under the supervision of adults who may be teachers or community volunteers [29,32].

This model aims to promote/encourage social interactions amongst students and with school staff, with the potential for learning about healthy eating through the use of Foodbank WA’s “Superhero Foods” concept and nutrition education resources [33]. The SBP also contributes to the development of food preparation skills. This is complemented by *Food Sensations*^®^, a practical, interactive nutrition education initiative aimed at educating and empowering children to make healthy life choices including how to choose and prepare healthier food [29,32]. The concepts delivered through *Food Sensations*^®^ can be delivered by teachers using age appropriate nutrition education resources and lesson plans developed by Foodbank WA. These resources are based on the Australian Guide to Healthy Eating [34] and mapped to the Australian national health curriculum. This is based on the premise that habits taught and learnt early in life are likely to be adopted and maintained into adulthood. More information about the programs offered by Foodbank WA is reported elsewhere [31].

Foodbank WA provides a range of shelf-stable food items at no charge to schools including canned fruit in juice, wheat biscuits, oats, vegemite, long life Ultra Heat Treated (UHT) milk (which can be stored without refrigeration before opening), canned spaghetti and baked beans [29,32]. Yoghurt, fresh bread and fresh fruit may be supplied to schools depending on the availability in the Foodbank WA stocks at the time of packing the orders. Registered schools are also able to access complementary health promotion initiatives through Foodbank WA’s “*Healthy Food for All*” strategy [29]. Schools applying to receive support from Foodbank WA are accredited through a process which involves provision of general guidelines regarding the minimum operational requirements including space, human resources and equipment needed to run a SBP [29]. Given the range of schools registered with Foodbank WA, from urban to remote setting, flexibility/autonomy occurs in how each individual school operates its SBP based on individual school needs. This flexibility recognizes that each school community has its own needs, and allows these to be taken in to account in actual program delivery for the SBP to be successful.

The variation in needs and social circumstances between different schools and communities has also been recognised during the implementation of SBPs. Some schools placed emphasis on simply providing a healthy early morning meal before the start of school, while others incorporated the elements of health education, encouraging social interaction between students and school staff, child care and developing basic skills in food preparation appropriate for age [35]. However, the UK now requires that food served in schools and academies in England under their jurisdiction meet the national school food standards so that children have healthy, balanced diets, and SBPs have been advocated for under the School Food Plan as an avenue for educating children, teachers and parents about good nutrition [36].

### 1.5. Background to the Current Project

Linked with research aimed at improving Indigenous education outcomes in two schools, it was observed that there were opportunities for improvement in the SBPs. The two principals of these schools, both of which had an existing SBPs, were approached and agreed to support a small project in their schools linked to the SBP. The project aimed at improving student’s understanding of nutrition, healthy eating and food origins and to identify possible improvements to the existing SBP model.

This paper describes the project which made assessment of SBP operation in two schools to highlight factors that hinder optimal performance of SBPs in the Midwest region of Western Australia (WA). It also describes the result of a purposeful intensive intervention to improve SBP functioning and operations in one school to improve on the delivery of the SBP and lessons learnt and offers recommendations that may be used to inform future policy and practice. While based on insights from working with only two schools, one based in a regional centre and the other in a remote town, the paper provides insights that have wider significance.

## 2. Methods

### 2.1. Setting

This project was undertaken in two schools in different rural settings in Western Australia. One was a primary school (Kindergarten to Year 6) located in a regional city of 30,000 people and within 5 km of a Foodbank of Western Australia (Foodbank WA) regional depot. The other was a District High School which has students from Kindergarten through to Year 12 and is located in a remote town over 300 km from the regional Foodbank depot, although core (non-perishable) products supplied to the remote school are transported out of Foodbank Perth. This reflects limited affordable transport options out of the regional city that services the region for the transport of bulk ambient (room temperature) product. Both schools have a high proportion of Aboriginal students, are located in areas of socioeconomic disadvantage and are part of the *More Than Talk* research project which is an Aboriginal-non-Aboriginal research partnership project which includes working with these two schools to improve Aboriginal education outcomes. There are other programs delivered by WA Centre for Rural Health within the schools as part of existing relationships where health science students undertake health-related service learning in the schools. It was while a nutrition student was observing the SBP at both schools that the need for further support to improve the nutritional component was identified. We consulted the principals and submitted an application for a small grant focused upon enhancing nutritional education in the schools and were successful in obtained funding.

### 2.2. Approach

Prior to commencement of the study described here, meetings were held with the respective school principals to discuss the purpose of the project, the planned evaluation and the intention for the findings to inform SBP operations state-wide. While the project was underway, regular meetings were held to update school stakeholders on the project progress and to provide opportunities for involvement and feedback.

Grey and peer-reviewed literature on models of SBPs implemented in the United States, United Kingdom and Australia were also reviewed, along with understanding the model of support offered by Foodbank WA and of the Healthy Food and Drink policy of the WA Department of Education [37] and described elsewhere [29,31]. This component of the project informed the inquiries to stakeholders and the approach adopted in the intervention.

### 2.3. Project Components and Data Collection

Stakeholder inquiry: Consultative meetings, informal interviews and discussions were held with stakeholders including the school principals, teachers, SBP volunteers and Foodbank WA staff, with observations and notes recorded. Participants described their experiences with and accounts of the operation of the SBP; their levels of satisfaction with the scheme; their observations of any changes to dietary intake, school behaviors and commented on children who regularly attend the breakfast sessions. We also asked for stakeholders’ views on the challenges faced running the SBP and areas/issues which they identified for improvements or change, and the nature of improvements they would like to see made.

Observations: Initially, we observed the SBPs in action on three separate occasions in each school, taking note of the menu, how food was stored, prepared and served, the level of student involvement in food preparation, the duration of the SBP, behavior and social interactions both amongst students and between staff and students. We noted the general set up, infrastructure, equipment, any information, education and communication materials available or used and general delivery of the breakfast programs. The regional school had an on-site vegetable garden which supplemented the SBP with fresh food supplies. As part of the observations we noted how the garden was used in the school and to what extent staff and students were involved in the garden activities. The remote school did not have a school garden, although it had run one previously.

School Breakfast Program Documentary review: We examined processes associated with ordering food from Foodbank WA and food delivery and reviewed invoices for food orders submitted to Foodbank WA against actual deliveries for a 6 month period (February to July 2016), noting the amounts ordered against the monthly maximum permitted for each item on the SBP order form. Stock shortages and excess stocks were noted and when this occurred we sought clarification from the school staff in-charge of ordering regarding any contingency arrangements when stock was unavailable. We also asked for the criteria/formulae used to calculate the food items requirements for orders, processes on food delivery to schools and requested information on the procedures the schools had in place around stock maintenance.

Summary Assessment: Using notes taken during meetings and interviews/discussions with key stakeholders, records from observations and analysis of documents, we generated detailed information about the operations, processes and key deliverables of the SBPs as well as the experiences of the various stakeholders involved in the delivery of SBP. This was reported back to school principals and Foodbank WA as key facilitators of the SBPs. We noted the programs’ strengths, system and structural factors that impeded realisation of optimal outcomes, and areas with potential for improvement.

Interventions: The researchers referred to published literature as well as proposals from stakeholders inform planning of suitable interventions .Interventions were implemented in a step-wise manner, as potential areas for improvement were identified.

Ethics Approval: Ethics approval for the *More Than Talk* was obtained from the WA Aboriginal Health Ethics Committee (367-10/11; 2015/671) and the University of WA. Approval for the research was provided by the WA Department of Education and both schools’ principals. All staff involved in data collection had necessary approvals to be in the schools. No formal data collection was undertaken with children.

## 3. Results

The two schools were selected based upon the previous agreement to be involved in the educational component of the *More Than Talk* education research which had a focus on exploring how existing evidence on best practice for improving educational outcomes was being implemented within the schools. As such, the principals had already committed to exploring relevant initiatives that include holistic support for Aboriginal students.

In describing the findings, we have integrated the results from all aspects of the inquiry into the SBPs. Of note was the consistency in the findings from triangulation of the different data sources.

### 3.1. Operations of the School Breakfast Program in Two Schools

Aspects of SBP delivery in the two schools are summarised in Table 1. In both schools, the SBPs ran every school day before the start of classes. Cereal, toast, spaghetti or baked beans, and milk, all supplied by Foodbank WA, were available. The remote school also had access to 100% unsweetened UHT orange juice and processed fruit juice with no added sugar owing to their isolation from Foodbank’s branch network (and corresponding inability to access fresh produce on a regular basis). The regional school also purchased sweetened jam, white bread and Milo from local stores using their own budget. There was little fruit served in either school, and manpower was an issue in the regional city with one ‘volunteer’ teacher rostered each day to working an extra hour in the morning before their routine teaching. Coordination was identified as suboptimal between the various departments involved in the running of the SBP within the regional school as well as between the school and other external stakeholders. No health education was evident during observation of both of the SBPs, and in the regional school no information, education and communication (IEC) materials were found within the SBP premises. While children were greeted by the staff supervising the SBP when they arrived, there as little encouragement of social interaction among the children or efforts that went beyond the transactional between the children and staff serving the food. In both schools children were not involved in any activity of food preparation. Stock shortages of some items occurred, particularly bread, spaghetti and baked beans whereas oats were overstocked in both schools.

Food ordering was not linked to calculations of average consumption rates in either school and the regional school did not have a system for documenting food order requests and deliveries. Parental involvement in the SBPs was minimal in both schools. Although the regional school had a school garden, it was run by only one class and teacher who felt there was little support provided from across the school in managing the garden. Other staff expressed that their involvement in the school garden was not welcomed. Fruit from Foodbank WA was provided to the regional school but was used in the *Crunch&Sip*^®^ program, and therefore was not available for the SBP.

These results informed the intervention implemented in the regionally based school as outlined below.

### 3.2. SBP Interventions in the Regionally-Based School

The initial assessment identified opportunities for improving the delivery of SBPs in the schools, as well as potential issues to be addressed. Each school had different requirements and areas to be addressed, therefore the intervention was tailored to the individual school. Recognising that holistic support that includes SBPs is a component of what has been shown to work in improving Aboriginal education outcomes, we were keen to use the SBP as an opportunity for improving nutrition understanding and habits and to develop a context for eating that would encourage social interaction and learning. As highlighted previously, evidence has shown that food, its consumption and the sharing of meals has positive social significance that exceeds its physical and nutritional value [21,26,38].

#### 3.2.1. Nutrition-Related Interventions

We discussed and proposed elimination from the menu of food items that did not comply with the Department of Education’s Healthy Food and Drink policy [37]. Through liaison and advocacy with Foodbank WA, an arrangement was made for a special fruit allocation to the SBP twice a week. We procured a juice maker so that fresh fruit and juice became a regular feature on the SBP menu. Efforts were made to build the capacity of school staff so as to increase their understanding and ability to calculate their average weekly and monthly consumption rates as the basis for making food order requests and to improve documentation of their orders and deliveries.

#### 3.2.2. Education-Related Interventions

A ten-week health education plan was developed and implemented using the Superhero Foods nutrition education resources supplied by Foodbank WA. Topics included some specific targeting of common unhealthy foods which children were likely to buy from the school canteen or have in their lunch boxes. Education sessions on healthy eating were undertaken during the SBP sessions twice a week and delivered in a fun and interactive way. Approaches included reading from the books of food characters, use of fruit and vegetable costumes, promoting engagement, learning and encouraging memory recall. During some sessions, a theme around particular fruit and where it came from was created as part of the session’s learning outcomes.

#### 3.2.3. Social-Interaction Related Interventions

Efforts were made to create an atmosphere that encouraged all children to socialise before food was served. When offered a more interactive and social environment, children enjoyed learning about food and reading aloud, even vying with each other to be selected to read. Using music and play helped settle the children. Involving children in decision-making around food and in the preparation of food and juice as appropriate for their age increased students’ active participation.

#### 3.2.4. Experience and Outcomes of the Regional Intervention

In the course of implementing the changes desirable from a public health perspective, it was evident that the interventions were not supported by all of the staff involved. Teachers who helped serve breakfast raised concerns that health education and social interaction were time-intensive activities and this encroached on their time for class preparation. This challenge could potentially be addressed by working to build the capacity of volunteers to deliver the health education sessions. Unfortunately for this school, there was only one external volunteer who had been committed to the program over many years and was away with illness over the full term when the intervention was initially implemented. Once she returned to her volunteer role, she was opposed to the health education sessions that had been incorporated into the SBP, evidently seeing the SBP function as about feeding children who would otherwise go hungry. Given resistance from the volunteer and rostered teachers that emerged, it was untenable to continue the enhanced SBP activities.

## 4. Discussion and Recommendations

Our engagement with these two SBPs arose as a result of the evidence that holistic support that includes SBPs as part of what works to enhance Aboriginal education outcomes [14]. The potential for SBPs to offer more than just nutrition for disadvantaged children has been well described elsewhere, and was reviewed as part of this study [26,38,39]. This paper critiques the operation of SBPs in two rurally-located schools and describes our experience in efforts to enhance the SBP offered in one regional school. Despite the limitations in scope of the project, the in-depth assessment of one regional school with more limited comparative assessment of a remote school provides insights that are important for how SBPs operate in disadvantaged schools more generally.

The findings and recommendations are summarised in Table 2.

### 4.1. Coordination Mechanisms

Establishing effective coordination mechanisms for the various actors involved in the delivery of SBPs, both those within and external to schools, is critical if SBP initiatives are to optimally meet the needs of disadvantaged children. While we were keen to enhance the holistic support provided by SBPs, we became aware of the complexity and challenges that occur with information flow between people within a school involved in the SBP, staff responsible for placing food orders to Foodbank WA and those who serve the breakfast, and which resulted in stocks being in under- and over-supply. Hence, oats accumulated because children preferred (or were only offered) other foods, yet orders continued. Those running the SBP reported having not had fresh fruit on the breakfast menu for months, yet once time was invested in understanding the system and resources available it was identified that fruit was available from Foodbank WA. We learnt fruit was being used instead in *Crunch&Sip*^®^, a program based upon having a set time during the school day to eat salad vegetables and fruit and to drink water in the classroom. This program usually requires students to bring vegetables or fruit to school each day for the *Crunch&Sip*^®^ break, yet for schools with many disadvantaged families this may be difficult to achieve.

Having effective coordination mechanisms with other external partners who can input into the various components of the SBP could also help optimise achieving an integrated and sustainable SBP that addresses the nutritional, educational and psychosocial needs of the children. Partnerships have particular importance when addressing complex issues and working with vulnerable populations [40,41]. It is also desirable that there is a forum that brings together all the main parties and stakeholders in each SBP. Such a forum could serve as an avenue for clarification and harmonising the services the SBP could provide in light of the circumstances of each school community (including human resources), enable discussion of facilitators and barriers to effective implementation and to identify practical strategies for implementation and sustainability. Such an approach could help in ensuring that the SBPs provide holistic services that meet the needs of disadvantaged children across multiple dimensions-psychosocial and educational—as well as putting food in their stomachs.

### 4.2. Human Resource Issues

Based on our experience in implementing a multi-faceted intervention in one school, it is evident that adequate human resources are crucial to the successful delivery of comprehensive services across the spectrum of nutrition, psychosocial support and nutritional education to children attending SBPs. Our experience suggests that schools under stress may tend to focus only on providing a healthy morning meal to students. It became clear that encouraging social interaction and provision of health education are time-intensive activities that place extra burden on the teachers helping to serve the breakfast who want that time to prepare for teaching. Ideally, SBPs would open earlier and close later than at present, increasing their duration and the time available for children to engage in social interaction and to receive educational messages regarding healthy food. Having committed volunteers inducted into a well-structured program would enable the SBPs to follow best practice approaches without competing for the teachers’ time for class preparation. A recent evaluation of SBPs in Victoria, a different socio-geographic context to that of rural WA, also indicated human resources to be a limiting factor; 50% of schools report staffing and sourcing volunteers was a significant barrier [42]. This report also suggested that schools with adequate staffing, along with Foodbank could share their knowledge and experience of successful techniques for recruiting and maintaining SBP volunteers with other schools.

The children who attend SBPs primarily come from parents of lower socio-economic status and can include families where both parents spend long hours at work. This means these parents are likely to have less time for interaction with their children and for preparing and supervising their children during meal times. This inevitably affects the quality of social interactions at home, so the informal social interaction networks that their children can develop in SBPs can contribute to developing secure relationships. Even in the context of the SBPs under consideration here, children often come from single parent homes where the carer has multiple competing demands on their time. External supports can also help build children’s resilience to many more adverse factors at home and in their lives that affect their educational outcomes. The opportunity to acquire skills and confidence around simple meals and recipes can then be put to use at home.

SBPs can play a role providing children from disadvantaged households with psychosocial nourishment and knowledge and education on healthy eating and can also model and encourage social interaction. Defeyter and colleagues found SBP attendance facilitated improvements in the quality of children’s relationships and was associated with a reduction in victimisation over time [22]. Although the regionally-based school employs a number of Aboriginal Education Assistants, they were not involved in the program. However, these staff could readily be trained and mentored in health education, and to encourage social interaction among the students and between the students and staff engaged in the SBP, using the nutrition education resources already developed by Foodbank WA. While they are a resource in high demand in the school, the SBP if delivered as a holistic program could help student interactions throughout the school and model the kind of interactions and relations with staff that have proved important in broader Aboriginal contexts [22,26,43,44,45]. In other Australian schools, senior students are involved in assisting their SBP as part of their role on the school council [42]. This has the double benefit of providing learning and leadership opportunities as well as helping staff the SBP.

It is important that any external partner that comes in to deliver the education and social interaction aspects to the SBP develops and maintains an effective working relationship with schools through means such as regular contact with all teachers and volunteers. Methods are needed to keep everyone involved up to date with relevant information and developments and enable opportunities to respond to any issues they raise. This is challenging within school environments, as it is desirable to avoid additional burden to school staff and recognise the efforts that staff make over and above core work requirements.

### 4.3. Food Standards/Menu

All food products supplied through Foodbank WA’s SBP comply with the Department of Education’s Healthy Food and Drink policy. It is critical that schools take responsibility to ensure that food sourced from non-Foodbank sources also complies, because of SBPs influence on children’s food preferences and choices. If the SBP menu is not generally meeting nutritional guidelines, it counteracts the intended objectives of providing a healthy morning meal and teaching students to make healthy food choices, with the aim of promoting healthy eating habits which are hoped to be long lasting. Story and colleagues describe the school as a setting that can have a large influence on a child’s eating behaviours [46]. SBPs are one part of the school environment, which collectively impacts children’s healthy eating habits and can thus affect future health outcomes. It is desirable that schools identify sources for and have contingency budgets that enable purchase of key perishable items for the breakfast menu-such as high fibre bread–and for those occasions when Foodbank WA does not have requested stock. It is also important that the school administrators take a keen interest in regularly scrutinising what is served to children in the program.

### 4.4. Logistics Management/Stocks Outs

In one school we were unable to access records of any invoices and delivery notes because there was no established filing system; copies of food provided needed to be obtained from Foodbank WA. Hence, we identified the need to establish systems to calculate average weekly and monthly consumption rates for the various food items and to use them for subsequent orders submitted to the Foodbank WA. Capacity building of staff to enable this could help minimise occurrences of running out of stocks and the accumulation of excess stock for some items. Staff were not fully aware of what food items were available from Foodbank WA, and we identified opportunities that had been missed to increase the supply of fresh fruit and vegetables for the regionally-based school. Improved understanding of the documentation and record keeping of food order invoices and delivery notes could improve the operation and functioning of SBPs and evaluation is an important element in efforts at service improvement.

### 4.5. Cold Storage

Limited storage of fresh foods in rural and remote areas of Australia has been shown to influence access to healthy foods [47]. As well as limitations in supermarkets located in these areas, this study has identified that storage is also a limiting factor in schools. Ensuring availability of adequate cold storage facilities would help to reduce on the frequency with which the schools have to pick perishable items from Foodbank WA depots. For remote schools it would be beneficial to establish connections with various regionally-based partners who have field officers who regularly travel to such remote towns to implement their activities and could assist with the delivery of perishable items, especially fresh fruit. This could ensure that the students in remote schools have a more regular supply of fresh fruit and also save the school the costs of freight for extra food items that run out during the course of the school term.

### 4.6. School Gardens

Establishment or access to a school garden can be beneficial to SBPs in that active participation in garden activities helps students to appreciate food origins and more about the processes that food goes through before it finally reaches the table. It also serves as a source of healthy foods to supplement those supplied by Foodbank WA. School gardens have been used as a component of nutrition education and shown to increase fruit and vegetable knowledge and cause behavior change among children [48,49]. However, the considerable work involved in developing and maintaining a school garden in the example of the regional school showed that it may result in issues related to ownership and access. Clear guidelines around school gardens are needed, and we suggest these should encourage their use as educational enhancement in SBPs as well as the use of produce there, and to ensure that school gardens benefit the wider school community.

## 5. Conclusions

SBPs are an important component of assisting children who for a multitude of reasons do not receive breakfast at home before coming to school. They serve as one element of holistic support for Aboriginal children and should assist their engagement with the school and improve their ability to learn. As well as feeding children, the children eagerly participated in social interaction and learning about nutrition, which may not be well modelled at home in areas of social disadvantage, where there is food insecurity. Such education, starting early, is particularly important given the high rates of chronic disease with particularly disproportionate rates of obesity, diabetes, cardiovascular disease occurring many years younger than for the general population in Australia [4,5]. All of these diseases necessitate the importance of effective nutrition interventions early in childhood, with schools an obvious setting. While the evidence for effective Aboriginal nutrition interventions is not strong, a review has found that community-based programs that engage and are led by members of the Aboriginal community and incorporate multiple components are most effective [50]. How best to involve Aboriginal parents and volunteers in SBPs needs further exploration.

Our work presents a more detailed assessment than that which has occurred through a survey evaluation of participating schools in the SBPs across WA, and which highlights that value of the SBP within schools and their ongoing commitment to it [32]. However, while SBPs have grown over many years and receive the bulk of their food free of charge through Foodbank WA [31], it is important to recognise that SBPs have opportunities to provide more than just food for children in socially disadvantaged areas and with fractured home environments. There are clearly challenges associated with asking busy teachers to do more volunteering and non-teaching duties outside of their teaching roles, yet based upon our “look through the keyhole” SBPs are not necessarily fulfilling their potential as a means to engage and build relationships with socially vulnerable children. Better engagement and support for community volunteers would seem an important strategy, and yet is particularly difficult in the disadvantaged environments which the schools examined in this study operate. However, it is important that the overall aim of an integrated SBP that addresses the nutrition, education and psychosocial needs of the children is realised in a way that ensures it is holistic and not paternalistic.

## Figures and Tables

**Table 1:** Summary of School Breakfast Program delivery in two WA schools, Term 3 2016.

Finding	Regional Based School	Remote Located School	Comment
Supported by Foodbank WA with healthy food items at no cost	Yes	Yes	
Purchases additional items from own budget	Yes	No	Regional school purchased sweetened jams, Milo and white bread
All items on menu comply with the Department of Education’s Healthy Food and Drink policy	No	Yes	The additional purchases of sweetened jams do not comply with healthy food policies.
Fruit included on breakfast menu	* No	No	* Fruit supplied by Foodbank WA was all used for *Crunch&Sip*^®^ program
Adequacy of human resources	No	Yes	Aboriginal Education Assistants support SBP in the remote school. Aboriginal education staff were not involved in the regional school
Volunteer support	Yes *	No	* One volunteer for 20 years, 2–3 days/week, Recent injury so absent for one term. (On return, the volunteer was resistant to program change)
Coordination structures with key stakeholders	Poor	Poor	No forum that brings together all stakeholders involved in the SBP
Usual duration of breakfast sessions in minutes	30	20	The approach feeds children as quickly as possible
Coordination within the school departments	* Poor	Good	* Volunteer coordinator on sick leave
Coordination with external stakeholders	Poor	Limited	
Information Education and Communication materials in place	Yes	No	Posters and Superhero resources
Health education undertaken	No	No	
Social interaction promoted	No	No	
Students involved in food preparation	No	No	Children were asked for what they want from the available options and have it prepared & given to them
Stock shortages reported	Yes	Yes	Fruit, bread, spaghetti, baked beans
Excess stocks reported	Yes	Yes	Oats
Documentation of orders and deliveries	Poor	Good	
Quantification of food orders based on sound criteria	Poor	Poor	
Adequacy of cold storage facilities	* No	Yes	* Fridge also used for teachers’ lunches
School garden in place	Yes	No	Not used for SBP
Level of students involvement in SBP garden	Minimal	N/A	
Parental/community involvement	No	Minimal	No focus on encouraging parental involvement

* further explanation of grading.

**Table 2:** Challenges and recommendations based upon findings.

Identified Challenge	Recommendations
***General***	
Lack of capacity–limited volunteer and teacher time	More buy-in is needed from the people running the program as well as more people involved in running the program. Explore avenues for recruiting more volunteers such as through increased involvement of parent associations.
Poor coordination and understanding of resources and support available from Foodbank WA	Strengthen coordination mechanisms. Improve communication between the various departments within the school and with other external stakeholders involved in the SBP by having regular review meetings.
Volunteers and staff running the SBP may have felt undervalued and under-supported to run a comprehensive SBP	Regular supervisory support from Foodbank WA to address issues through mentoring new staff in schools and providing timely feedback on common operational challenges that schools face. Time allocated to staff involved in SBP needs to be recognised and valued. Ideally such time should reduce teaching duties.
Develop clear objectives of the program which recognise the role of the SBP as more than just providing food	Educate and support staff and volunteers to understand the multiple potential roles of the SBP which include holistic support beyond providing food: potential benefits on children’s health include on cognitive performance and academic attainment as well as facilitating interaction and modelling social skills to children.
***Nutrition and Food Storage***	
Lack of adherence to Schools Food and Drink policy	Education of staff and volunteers regarding the policy and the importance of schools in establishing and modelling good eating patterns to children.
Poor stock maintenance resulting in shortage or oversupply of staple food items.	Better induction and training of school staff on policies and procedures of Foodbank WA and on ordering and stock management. Schools identify a contingency budget for purchasing some food items that are unable to be supplied by Foodbank WA. Schools should explore arrangements with local growers/shop owners with respect to support for the SBP.
Dual use of a fridge for storage of SBP food and staff lunches	A dedicated fridge is required for SBP and could assist with maintenance and inventory of stock. This is particularly important if there are multiple helpers involved.
***Education***	
Failure to embrace the SBP as a potential opportunity for health education; SBP currently seen as a non-teaching volunteer role.	Quality induction of all volunteers and staff involved in the SBP to convey the importance of educating children about healthy eating.
The food and nutrition resources provided by Foodbank WA are underutilised	Ensure those running SBPs know how to use the Superhero Foods resources to support children’s education and learning.
No education about ‘paddock to plate’	Embrace opportunities to learn about growing and producing food, engagement with the school garden.
***Social interaction***	
Low community volunteer support for the SBP	Encourage parental involvement in school nutrition and cooking programs, including volunteering in the SBP. Strengthen community engagement and involvement through activities such as establishment and support of school community gardens.
Inadequate attention to SBPs as being welcoming and engaging	Reimagining SBPs as places for building social connections for vulnerable children-places for laughter, music, learning and friendships.
Strengthen involvement of Aboriginal people supporting the program	Build the capacity of Aboriginal and Torres Strait Islander education officers/assistants. Explore and implement opportunities for Aboriginal parental involvement in learning about nutrition and supporting SBPs. Introduce cultural story telling in relation to traditional foods could help engagement.
